# OTME-17. Single cell characterization of the immune microenvironment of melanoma brain and leptomeningeal metastases

**DOI:** 10.1093/noajnl/vdab070.068

**Published:** 2021-07-05

**Authors:** Inna Smalley, Zhihua Chen, Manali Phadke, Jiannong Li, Xiaoqing Yu, Clayton Wyatt, Brittany Evernden, Jane Messina, Amod Sarnaik, Vernon Sondak, Chaomei Zhang, Vincent Law, Nam Tran, Arnold Etame, Robert Macaulay, Zeynep Eroglu, Peter Forsyth, Paulo Rodriguez, Ann Chen, Keiran Smalley

**Affiliations:** Moffitt Cancer Center, Tampa, FL, USA

## Abstract

Melanoma brain metastases (MBM) and leptomeningeal metastases (LMM) are two manifestations of melanoma dissemination to the CNS with vastly different survival outcomes. Analysis of single cell RNA-Seq data from 43 clinical specimens has uncovered a distinct, immune-suppressed T cell landscape in the LMM microenvironment that is distinct to those of the brain and skin metastases. An LMM patient with an extraordinarily long survival and documented response to therapy demonstrated an immune repertoire that was distinct from those of typical poor survivors and more similar to CSF from non-LMM donors. Analysis of serial specimens over the course of therapy demonstrated reductions in melanoma cells and macrophages, coupled with increased levels of T cells and dendritic cells in the CSF of the extraordinary responder, whereas poor survivors showed no improvement in T cell responses. In MBM patients, targeted therapy and immunotherapy was associated with increased immune infiltrate, with similar T cell transcriptional diversity noted between skin metastases and MBM - suggestive of immune cell trafficking into the brain. Treatment with targeted therapy was associated with an enrichment of CD8 T cells. Immunotherapy was associated with a more diverse lymphocyte landscape and higher numbers of antibody-producing cells. These findings were confirmed by multiplexed staining of patient specimens and using an immune-competent mouse model of MBM. Correlation analysis across the entire immune landscape identified the presence of a rare, novel population of dendritic cells (DC3s) to be correlated with increased overall survival, regardless of disease site/treatment. The presence of DC3s positively regulated the immune environment of both patient samples and preclinical melanoma models through modulation of activated T cells and MHC expression in the tumor. Our study provides the first comprehensive atlas of two distinct sites of melanoma CNS metastases and identifies rare populations of cells that underlie the biology of this devastating disease.

